# A Bayesian Integrative Mixed Modeling Framework for Analysis of the Multi-Site Adolescent Brain and Cognitive Development Study

**DOI:** 10.1080/26941899.2025.2600125

**Published:** 2025-12-23

**Authors:** Aidan Neher, Apostolos Stamenos, Mark Fiecas, Sandra E. Safo, Thierry Chekouo

**Affiliations:** Division of Biostatistics and Health Data Science, School of Public Health, University of Minnesota, Minneapolis, MN, USA

**Keywords:** Neuroimaging, early life adversity, latent variables, predictive modeling, multi-view integration, hierarchical bayesian factor models

## Abstract

Integrating high-dimensional, heterogeneous data from multi-site cohort studies with complex hierarchical structures poses significant variable selection and prediction challenges. We extend the Bayesian Integrative Analysis and Prediction (BIP) framework to enable simultaneous variable selection and outcome modeling in data of a multi-view nested hierarchical structure. We apply the proposed Bayesian Integrative Mixed Modeling (BIPmixed) framework to the Adolescent Brain Cognitive Development (ABCD) Study, leveraging multi-view data, including structural and functional MRI and early life adversity (ELA) metrics, to identify relevant variables and predict the behavioral outcome. BIPmixed incorporates 2-level nested random effects to enhance interpretability and make predictions in hierarchical data settings. Simulation studies illustrate BIPmixed’s robustness in distinct random effect settings, highlighting its use for complex study designs. Our findings suggest that BIPmixed effectively integrates multi-view data while accounting for nested sampling, making it a valuable tool for analyzing large-scale studies with hierarchical data.

## 1. Introduction

Motivated by the Adolescent Brain and Cognitive Development (ABCD) Study, the largest long-term study of brain development in the United States (Garavan et al. 2018; Volkow et al. 2018; Saragosa-Harris et al. 2022), we extend a multi-view learning method, where multiple views or data types are integrated into one analysis, to multi-site studies with hierarchically nested data. The ABCD Study has a hierarchically nested design; specifically, individuals are nested within families, which in turn are nested within a study site. In addition, the ABCD Study contains high-dimensional data from heterogeneous views (e.g. brain imaging, mental health & substance use screenings, physical health measurements) on up to 90K variables, excluding SNP data, on each of the 11,878 subjects (Saragosa-Harris et al. 2022). The ABCD Study has been used to understand diverse phenomena, including Early Life Adversity (ELA): The experience of negative events early in life that can impact a youth’s developmental trajectory (Brieant et al. 2023; Orendain et al. 2023).

High-dimensional heterogeneous views make manual identification of relevant variables labor-intensive and prone to differential curation. This is especially true for analyses of ELA since its broad definition means that it can be defined by a large collection of variables (Brieant et al. 2023; Orendain et al. 2023). In this paper, we will integrate multiple brain imaging data types with ELA measures and identify the relevant variables predictive of externalizing problems. Modern research is moving toward a data-driven understanding of psychopathology (Kotov et al. 2021; Cuthbert 2022; Conway et al. 2023). Following this trend, our work aims to identify the variables and pathways that contribute to externalizing behaviors in adolescents. We develop a Bayesian mixed modeling framework (BIPmixed) that accounts for the ABCD Study’s nested sampling design while performing variable selection and outcome modeling.

BIPmixed is a form of Bayesian multi-view supervised learning, which means that BIPmixed uses a Bayesian framework to integrate multiple views (i.e. data types) in the prediction of an outcome y. A naive form of multi-view supervised learning is to concatenate views and use the larger matrix to predict some outcome vector y. An alternative, though also naive, approach is to fit predictive models from each view to the outcome y and, in a second step, combine the resulting predictions $\overset{ˆ}{y}$ by taking the mean, for example. The former is “early fusion” and the latter “late fusion”. These approaches are naive in that they do not account for the underlying structure and relationships between views in modeling the outcome. In contrast to early and late fusion, supervised intermediate fusion methods have been proposed to simultaneously estimate shared latent structure in views and model the outcome of interest, and BIPmixed is a supervised intermediate fusion method. Hybrid methods, such as cooperative learning (Yi Ding et al. 2022), have also been proposed to combine early and late fusion methods. Two-step multi-view learning algorithms are an alternative to the approaches mentioned above. These methods primarily focus on the first step of dimension reduction of multiple views into only shared (i.e. joint) structure (Argelaguet et al. 2018, 2020), or both shared and view-specific structure (Shen et al. 2009, 2013; Lock et al. 2013). This can be thought of as an integrative or multi-view factor analysis. In the second step, outputs from multi-view factor analyses are usable in an outcome model. This approach shares similar issues with early and late fusion, in that the outcome y is not included in latent structure estimation and variable selection; therefore, results from this approach cannot be interpreted directly in connection to the outcome. Thus, we focus on supervised integrative (or one-step) fusion methods, either intermediate or hybrid.

Several supervised integrative methods, from frequentist and Bayesian perspectives, have been developed to simultaneously integrate multiple views and an outcome y. Frequentist methods include CVR (Luo et al. 2016), DIABLO (Singh et al. 2019), JACA (Zhang and Gaynanova 2018), deep IDA (Wang and Safo 2024), and SIDA (Safo et al. 2022). Bayesian integrative and outcome modeling methods for joint variable selection and prediction include (Yuan et al. 2011; Klami et al. 2013, 2015; Wang et al. 2013; Chekouo et al. 2017; Mo et al. 2018; Chekouo and Safo 2022). While these methods have shown promise in the literature, a key limitation is their inability to account for the hierarchically nested structure typical of large cohort studies, such as our motivating ABCD Study. If observations from the same node of a hierarchy (e.g. subjects from a family in the same research site) are correlated but are treated as independent units, we limit our inferential capacity.

We extend Bayesian Integrative analysis and Prediction (BIP) (Chekouo and Safo 2022), which is a supervised intermediate fusion framework that allows simultaneous view integration and outcome modeling. Furthermore, BIP allows for variable selection and the incorporation of prior knowledge. Our extension, BIPmixed, builds on the strengths of BIP but also accounts for the hierarchically nested data that characterizes the ABCD Study and is typical of large cohort studies. Also, in BIPmixed, (clinical) covariates (e.g. sex, age, BMI, etc.) are included in the outcome model, rather than as a separate view, which can be useful when covariates are strongly predictive of the outcome (Cheng et al. 2016). In Section 2, we describe the ABCD Study dataset and our proposed BIPmixed approach. In Section 3, the ABCD application’s results show that including covariates as fixed effects in the BIPmixed outcome model yielded a 4% improvement in predictive accuracy (lower test MSE) and a 12% improvement in stability (lower SD of test MSE) over BIP, while joint variable selection isolated two outcome-linked latent components that co-localized key ELA indicators with sMRI thickness/surface area and fMRI connectivity features. In Section 4, simulations confirm that BIPmixed recovers latent structure shared across views and has greater predictive accuracy in hierarchically structured data relative to comparators. Finally, we discuss strengths and limitations in Section 5 and conclusions in Section 6.

## 2. Methods

### 2.1. ABCD Study and Motivating Scientific Question

The ABCD Study is the largest study of brain development. With modern psychopathology research becoming more data-driven, we aim to understand behavioral dysregulation *via* a statistical framework that integrates the ABCD Study’s heterogenous views (e.g. brain imaging and survey data) and accounts for the ABCD Study’s hierarchically nested structure (families in study sites). Specifically, the scientific question is: Which aspects of early life adversity (ELA) and brain structure and function are most strongly associated with externalizing problems in early adolescence? To address this, we integrate data modalities—including survey-based ELA measures, structural MRI (surface area and cortical thickness), and resting-state functional connectivity—in the context of a mixed model to identify variables jointly informative of behavioral dysregulation reflected in externalizing problem scores.

**Figure 1.**
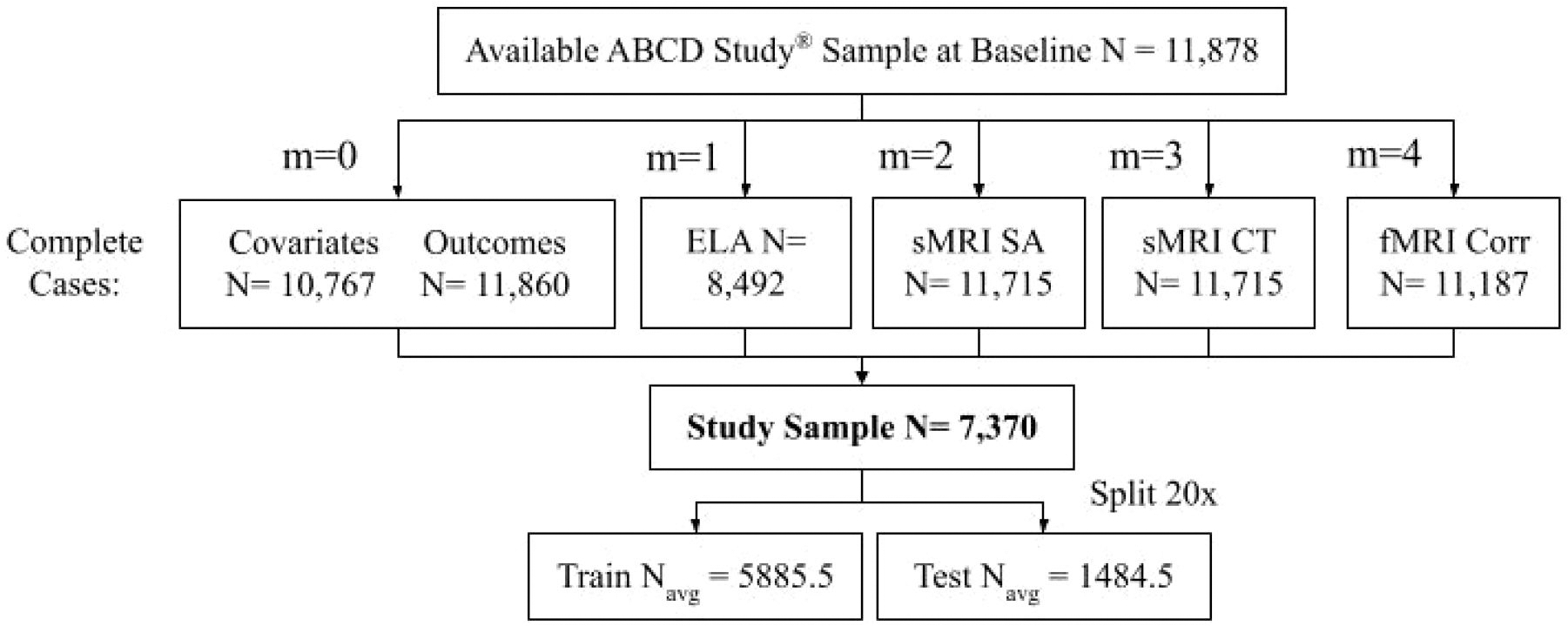
Flowchart of sample selection from the ABCD Study at baseline (*n* = 11,878). Subjects excluded for missing any data in each view, Early Life Adversity (ELA), structural MRI Surface Area (sMRI SA), structural MRI Cortical Thickness (sMRI CT), functional MRI correlations between Gordon Atlas Regions of Interest (fMRI Corr), covariates, and outcomes variable Externalizing Problems. Resulting sample size *n* = 7,370. On stratifying by study site, data is repeatedly split into 20 train and test sets with an 80:20 ratio respectively, which results in train $N_{\mathrm{avg}}=5885.5$ and test $N_{\mathrm{avg}}=1484.5$.

We performed a cross-sectional analysis using the baseline from the 5.1 data release. Our analysis includes the behavioral outcome externalizing problems as raw scores obtained from the Child Behavior Checklist (Achenbach and Ruffle 2000)) (where higher scores correspond to elevated levels of exteneralizing behaviors), covariates (e.g. sex), and four views (M=4): Early Life Adversity (ELA), structural MRI Surface Area (sMRI SA), structural MRI Cortical Thickness (sMRI CT), functional MRI correlations between Gordon Atlas Regions of Interest (fMRI Corr). Figure 1 describes the contribution of each of these data to our analysis sample. We describe the outcome externalizing problems and the covariates of the selected sample in Table 1 and explore the impact of covariate inclusion on prediction by performing the analysis with and without covariates included in the modeling.

To study the ELA view, we used the union of variables from two ELA studies (Brieant et al. 2023; Orendain et al. 2023). With ELA variables representing survey responses, some variables have disproportionately high rates of non-response, which we define as an individual not answering the survey question whatsoever, or extremely low rates of endorsement, which is a preponderance of individuals responding with “no”. We excluded ELA variables with >50% non-response and <0.05% endorsement. Endorsement filtering resulted in dropping 1 variable from Orendain et al. 2023. These decisions led to an ELA view of 88 variables. We included structural MRI (sMRI) metrics as 2 different views: cortical surface area (sMRI SA) and cortical thickness (sMRI CT), parcellated by the Destrieux Atlas, which has 74 regions of interest (ROIs). With 74 ROIs across 2 hemispheres, we had 148 variables in sMRI CT and sMRI SA, respectively. Resting-state functional MRI (fMRI) connectivity between ROIs from the Gordon Network is the 4th view. Connectivity measures included pairwise and self-correlations between 13 networks, resulting in 13^2^ = 169 variables.

Subjects are excluded for missing data in any view, covariates, and externalizing problems, leading to *n* = 7,370 (Figure 1). The ABCD Study is hierarchically nested in that subjects with siblings in the study are nested in families in 22 study sites across the United States, including, for example, Children’s Hospital Los Angeles, Florida International University, and the Laureate Institute for Brain Research (LIBR). By accounting for the nested design, the identification of ELA and brain variables associated with externalizing problems becomes more rigorous and interpretable. Additionally, on stratifying by study site, data is repeatedly split into 20 train and test sets with an 80:20 ratio respectively, which results in train $N_{\mathrm{avg}}=5885.5$ and test $N_{\mathrm{avg}}=1484.5$ with which we evaluate the Mean Square Prediction Error (MSE).

### 2.2. Mixed Outcome Model

Outcome y in the data analysis is the behavioral outcome externalizing problems. In the outcome model, fixed effects β correspond to the design matrix W, which reflect, for example, age and sex in the ABCD Study. Random effects θ are included by the design matrix Z, to account for the families nested in study sites. At the individual observation level, for individual i in family f within site s, the mixed outcome model is defined as

$$y_{i}=y_{ifs}=W_{i}\beta +Z_{i}\theta +U_{i}\alpha +\varepsilon_{i}=W_{i}\beta +\theta_{f:s}+U_{i}\alpha +\varepsilon_{i},$$

where $U_{i}$ is the i th row of a factor that represents latent structure shared across M views—as defined in Section 2.3.

The outcome view’s loadings α follow a spike and slab prior distribution: $a_{l}|\gamma_{l}\sim \left(1-\gamma_{l}\right)\delta_{0}+\gamma_{l}N\left(0,\tau_{l}^{2}\sigma^{2}\right)$. $W_{i}$ is the i th row from the fixed effect design matrix of dimension $p_{\beta }$, the number of covariates. β is the fixed effects coefficient vector of length $p_{\beta },\;\theta_{f:s}$ is the contribution of family f in site s to the intercept, and $\varepsilon_{i}=\varepsilon_{ifs}|\sigma^{2}\overset{iid}{\sim }𝒩\left(0,\sigma^{2}\right)$. Priors for the fixed and random effects are specified as follows: $\beta \sim 𝒩(0,\sigma_{\beta }^{2}I_{p_{\beta }})$; $\mu \sim 𝒩\left(0,\sigma_{\mu }^{2}\right)$; $\xi_{s}|\mu ,\sigma_{\xi }^{2}\overset{iid}{\sim }𝒩(\mu ,\sigma_{\xi }^{2})$; $\theta_{f:s}|\xi_{s},\;\sigma_{\theta_{s}}^{2}\overset{iid}{\sim }𝒩(\xi_{s},\sigma_{\theta_{s}}^{2})$; $\sigma_{\xi }^{2}\sim IG(a_{\xi },b_{\xi })$; $\sigma_{\theta_{s}}^{2}\overset{\mathrm{iid}}{\sim }IG(a_{\theta },b_{\theta })$; $\sigma^{2}\sim IG\left(a_{\sigma },b_{\sigma }\right),\;$ where IG(a,b) is inverse gamma with the shape parameter a and scale b. We can interpret μ as the grand mean or intercept and $\xi_{s}$ as the site s effect, which centers the $\theta_{f:s}$ family in site effects. Variance parameters $\sigma_{\beta }^{2},\;\sigma_{\mu }^{2},\;\sigma_{\xi }^{2}$, and $\sigma_{\theta_{s}}^{2}$ represent the fixed effect variability, that in the grand mean, the site effects, and family in site effects, respectively. We let $\sigma_{\theta }^{2}=(\sigma_{\theta_{1}}^{2},\ldots ,\sigma_{\theta_{s}}^{2})$ as the vector of site-specific variances. Section 2.8 details hyperparameter specification.

### 2.3. Multi-View Factor Analysis Framework

**Figure 2.**
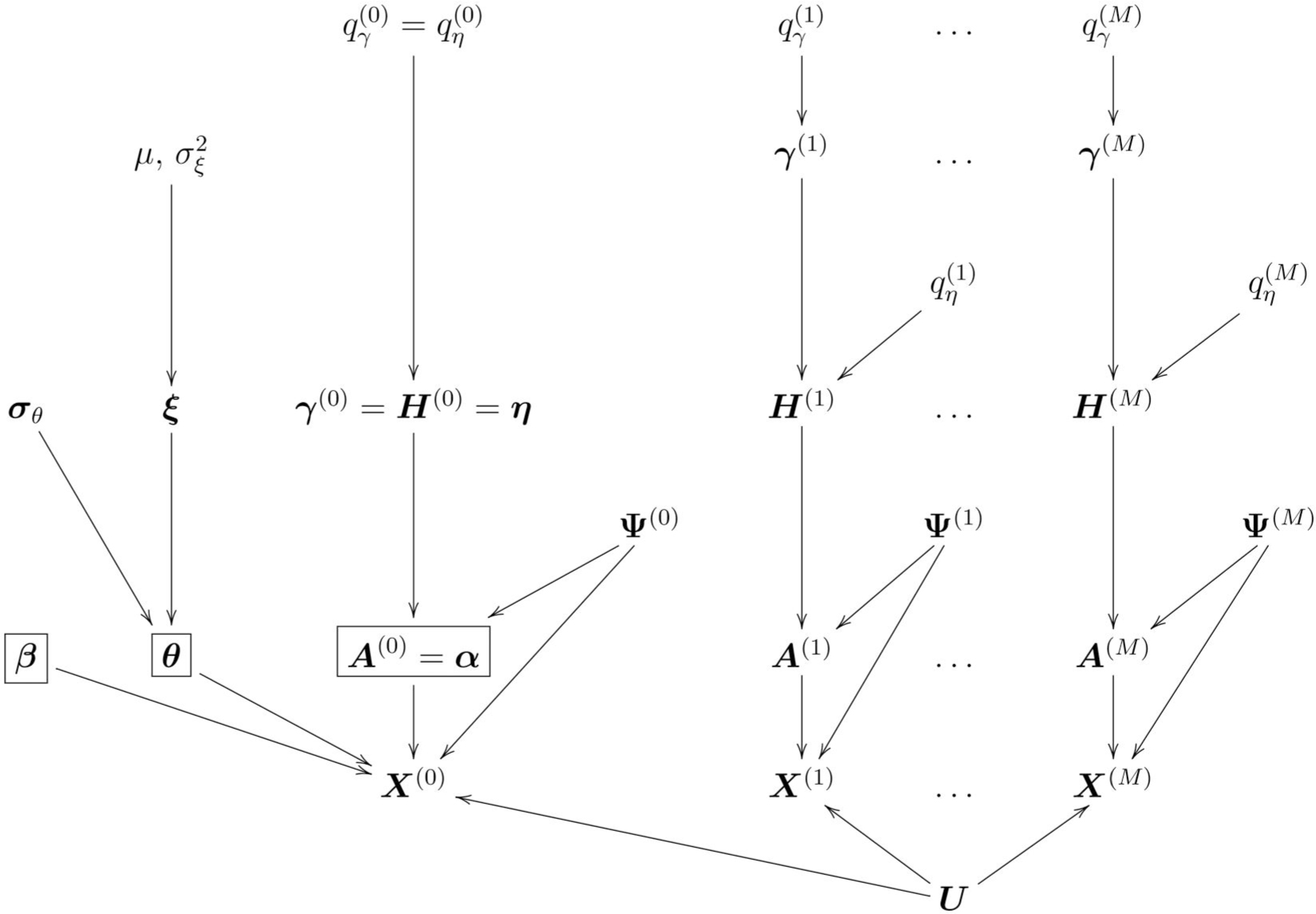
Graphical representation of the proposed probabilistic model that connects the M+1 views. This method integrates M+1 data views comprised of the outcome of interest y and other views (e.g. imaging, or multi-omics data). The correlation between different views is modeled through the shared matrix U, and individual sources of variation are modeled through the individual loading matrices $A^{\left(m\right)}$ for m∈{0,1,…,M}. Binary vectors and matrices $\gamma^{\left(m\right)},\;H^{\left(m\right)}$ are incorporated for variable and latent component selection. In addition to the variable/component selection indicators, the outcome view $X^{\left(0\right)}$ also incorporates fixed effects β as well as hierarchically nested random effects θ and ξ.

In Figure 2, we describe a Bayesian factor analysis framework for integrating information across M views of observed data. For each view m=1,…,M, the observed data matrix is $X^{\left(m\right)}=UA^{\left(m\right)}+E^{\left(m\right)}$, where $X^{\left(m\right)}\in R^{n\times p_{m}},\;n$ is the number of observations, and $p_{m}$ is the number of features in view m. The latent factor matrix is $U\in R^{n\times r}$, where each row $U_{i}\overset{iid}{\sim }{𝒩}_{r}\left(0,I_{r}\right),\;i=1,\ldots ,n$, represents an r-dimensional latent factor vector for subject i. The loading matrix for view m is $A^{\left(m\right)}=(a_{lj}^{\left(m\right)})\in R^{r\times p_{m}}$, which maps latent factors into the feature space of view m. The noise matrix is $E^{\left(m\right)}\in R^{n\times p_{m}}$, where each row $E_{i}^{\left(m\right)}\overset{\mathrm{iid}}{\sim }{𝒩}_{p_{m}}(0,Diag(\sigma_{1}^{2(m)},\ldots ,\sigma_{p_{m}}^{2(m)}))$ is $p_{m}$-dimensional noise vector for subject i.

### 2.4. Variable and Latent Component Selection

We aim to identify a set of variables that are associated across views and biologically interpretable. As in BIP (Chekouo and Safo 2022), we introduce the binary indicator variables: $\eta_{lj}^{\left(m\right)}$ is a binary variable selection indicator, and $\gamma_{l}^{\left(m\right)}$ is a latent factor component selection indicator. For outcome view $X^{\left(0\right)},\;\eta_{l1}^{\left(0\right)}=\gamma_{l}^{\left(0\right)}$. Prior for $\eta_{lj}^{\left(m\right)}$ is a mixture between a point mass at 0, $\delta_{0}$ (indicating variable j excluded in l th latent component) and $Bernoulli\left(q_{\eta }\right)$, and $\gamma_{l}^{\left(m\right)}$ is Bernoulli $\left(q_{\gamma }\right)$. Hyperparameters $q_{\eta }$ and $q_{\gamma }$ are prior probabilities to select variables and components, respectively. Factor loading prior to $a_{lj}^{\left(m\right)}$ depends on both $\gamma_{l}^{\left(m\right)}$ and $\eta_{lj}^{\left(m\right)}$. The prior is a mixture of $\delta_{0}$ and a normal distribution where $\tau_{lj}^{2}$ is a shrinkage parameter: $a_{lj}^{\left(m\right)}|\gamma_{l}^{\left(m\right)},\eta_{lj}^{\left(m\right)}\sim (1-\gamma_{l}^{\left(m\right)}\eta_{lj}^{\left(m\right)})\delta_{0}+\gamma_{l}^{\left(m\right)}\eta_{lj}^{\left(m\right)}𝒩(0,\tau_{lj}^{2}\sigma_{j}^{2(m)})$. If, for example, the l th latent component indicator $\gamma_{l}^{\left(m\right)}$ or the indicator for the j th variable in the l th component $\eta_{lj}^{\left(m\right)}$ is equal to 0, then the loading for the j th variable in the l th component is set to 0 with probability 1. Otherwise, we have normal loadings that have variance dependent on $\tau_{lj}^{2}$ and $\sigma_{j}^{2(m)}$.

### 2.5. Prediction

We predict for a new set of individuals that belong to a known site s with $X_{\mathrm{new}}^{\left(m\right)},\;m=1,\ldots ,M$ observed. Given a model $\left\{\gamma^{\left(m\right)},H^{\left(m\right)}:m=0,1,\ldots ,M\right\}$ which consists of the set of latent component selection indicator vectors and variable selection matrices, respectively, we estimate loadings as ${\overset{ˆ}{a}}_{.j(\eta )}^{\left(m\right)}={\overset{ˆ}{\sigma }}_{j}^{2(m)}{\left({\bar{U}}_{\left(\gamma \right)}^{T}{\bar{U}}_{\left(\gamma \right)}+I_{n_{\gamma }}\right)}^{-1}{\bar{U}}_{\left(\gamma \right)}^{T}x_{.j}^{\left(m\right)},\;$ where posterior mean estimates ${\overset{ˆ}{\sigma }}_{j}^{2(m)}$ and $\bar{U}$ are used, and subscripts (γ) and (η) indicate elements were $\gamma_{l}=1$ or $\eta_{lj}=1$. For the outcome model loadings, we let $x_{.j}^{\left(0\right)}=y-W\overset{ˆ}{\beta }-Z\overset{ˆ}{\theta }$ where $\overset{ˆ}{\beta }$ and $\overset{ˆ}{\theta }$ are posterior mean estimates for obtaining ${\overset{ˆ}{a}}_{.j(\eta )}^{\left(0\right)}={\overset{ˆ}{\alpha }}_{\left(\gamma \right)}$. We use $\overset{ˆ}{\xi }$ as an estimate for $\overset{ˆ}{\theta }$ since the family effect in the test set has not been observed. From ${\overset{ˆ}{A}}_{\left(\eta \right)}=({\overset{ˆ}{a}}_{j(\eta )}^{\left(1\right)},\ldots ,{\overset{ˆ}{a}}_{.j(\eta )}^{\left(M\right)})\;$ and $X_{\mathrm{new}\;}=(X_{\mathrm{new}}^{\left(1\right)},X_{\mathrm{new}}^{\left(2\right)},\ldots ,X_{\mathrm{new}}^{\left(M\right)})$, both column-wise concatenated matrices, and $D\left({\overset{ˆ}{\sigma }}^{-2}\right)$, a diagonal matrix with posterior means ${{\overset{ˆ}{\sigma }}_{j}}^{2(m)},\;m=1,\ldots ,M,\;j=1,\ldots ,p_{m}$ as elements, we estimate ${\overset{ˆ}{U}}_{\mathrm{new,}(\gamma )}=({\overset{ˆ}{A}}_{\left(\eta \right)}D\left({\overset{ˆ}{\sigma }}^{-2}\right){\overset{ˆ}{A}}_{\left(\eta \right)}^{T}+{\left.I_{r}\right)}^{-1}{\overset{ˆ}{A}}_{\left(\eta \right)}D\left({\overset{ˆ}{\sigma }}^{-2}\right)X_{\mathrm{new}}$. With $W_{\mathrm{new}}$ and $Z_{\mathrm{new}}$ for new individuals’ fixed and random effect design matrices, we have, for 1 model, ${\overset{ˆ}{y}}_{\mathrm{new}}={\overset{ˆ}{U}}_{\mathrm{new},(\gamma )}\alpha_{\left(\gamma \right)}+W_{\mathrm{new}}\overset{ˆ}{\beta }+Z_{\mathrm{new}}\overset{ˆ}{\theta }$. From Bayesian model averaging over a maximum number of models, which are selected as the models with largest posterior probability, the prediction of new subjects is estimated as: ${\overset{ˆ}{y}}_{\mathrm{new}}=W_{\mathrm{new}}\overset{ˆ}{\beta }+Z_{\mathrm{new}}\overset{ˆ}{\theta }+\sum_{\eta ,\gamma }{\overset{ˆ}{U}}_{\mathrm{new},(\gamma )}\alpha_{\left(\gamma \right)}p(\gamma ,\eta |{\bar{U}}_{\left(\gamma \right)},X)$.

### 2.6. Competing Methods

We compare BIPmixed against the original BIP framework, which does not account for random effects, in both the ABCD Study data analysis and the simulation studies (Chekouo and Safo 2022). In the simulation study, we also evaluate performance in simulations against Cooperative Learning Lasso with a hyper-parameter ρ=0.5. i.e. halfway between early and late fusion, using 10-fold cross-validation (CV) on the training set to choose the penalty parameter (Ding et al. 2022). Furthermore, we include a 2-step method: PCA2Step, which does not account for the multiview data structure. PCA2Step starts with Principal Components Analysis of concatenated train set views, and then, in the second step, the top 4 principal components are used in a frequentist linear mixed model—where 4 latent factor components are used in data generation.

### 2.7. Evaluation Criteria

Across the S datasets, we evaluate variable selection and prediction performance. Using a Marginal Posterior Probability (MPP) threshold of 0.5, we calculate the false positive rate (FPR) and false negative rate (FNR). Additionally, to evaluate variable selection in a threshold-independent way, we estimate the AUC of variable classification as important or not. AUC of variable selection probabilities is not available for methods that only support binary variable selection, e.g. Cooperative Learning. Prediction metrics are mean square error (MSE) and $Var(\overset{ˆ}{y})$ and are computed for all the methods in the test data.

### 2.8. Hyperparameter Specification

The loadings shrinkage parameter $\tau_{lj}^{2}$ is fixed to 1. Its properties are explored more in Chekouo et al. (2017). The maximum number of models in Bayesian Model Averaging (BMA) prediction is 50, since more models give similar results. Non-outcome view variable selection prior parameter $q_{\eta }$ set to 0.05 by the assumption of sparse variable importance. The prior probability of latent factor component selection $q_{\gamma }$ was assigned a Beta(1,1) distribution, representing a uniform prior with no preference toward inclusion or exclusion. Fixed effect vector β has an uninformative normal prior with uncorrelated fixed effects, the same variance $\sigma_{\beta }^{2}$ fixed at a large value of 100, and prior mean 0. Other priors’ hyperparameters are set uninformatively, and particular values are available as default arguments in the BIPmixed implementation from the Code Availability Section 8.1.

We choose minimally a latent component number r equal to the number of views, including the outcome view, M+1, to be analyzed such that every component can be associated with only one view. For example, if gene expression and SNP data are included as 2 views in modeling outcome y, let $r_{\min}=3$. To get a preliminary understanding of the data’s latent structure, we can concatenate the non-outcome views and y column-wise, standardize each column to mean zero and variance one, and then calculate the sample covariance matrix and its associated eigenvalues. After inspecting a scree plot, we can choose r to be where the eigenvalues stabilize begin to level off. For the data analysis, to choose r - the number of latent factors - we compute the covariance matrix by first concatenating the 4 views, the outcome externalizing problems, and the covariates column-wise. We standardize so that each variable has mean zero and variance one and calculate the covariance matrix, and its associated eigenvalues to choose hyperparameter r=6 where the eigenvalues level off (Figure 5). For the simulation study, we fix r=4, which is the same as the number of views.

## 3. Data Analysis Results

**Figure 3.**
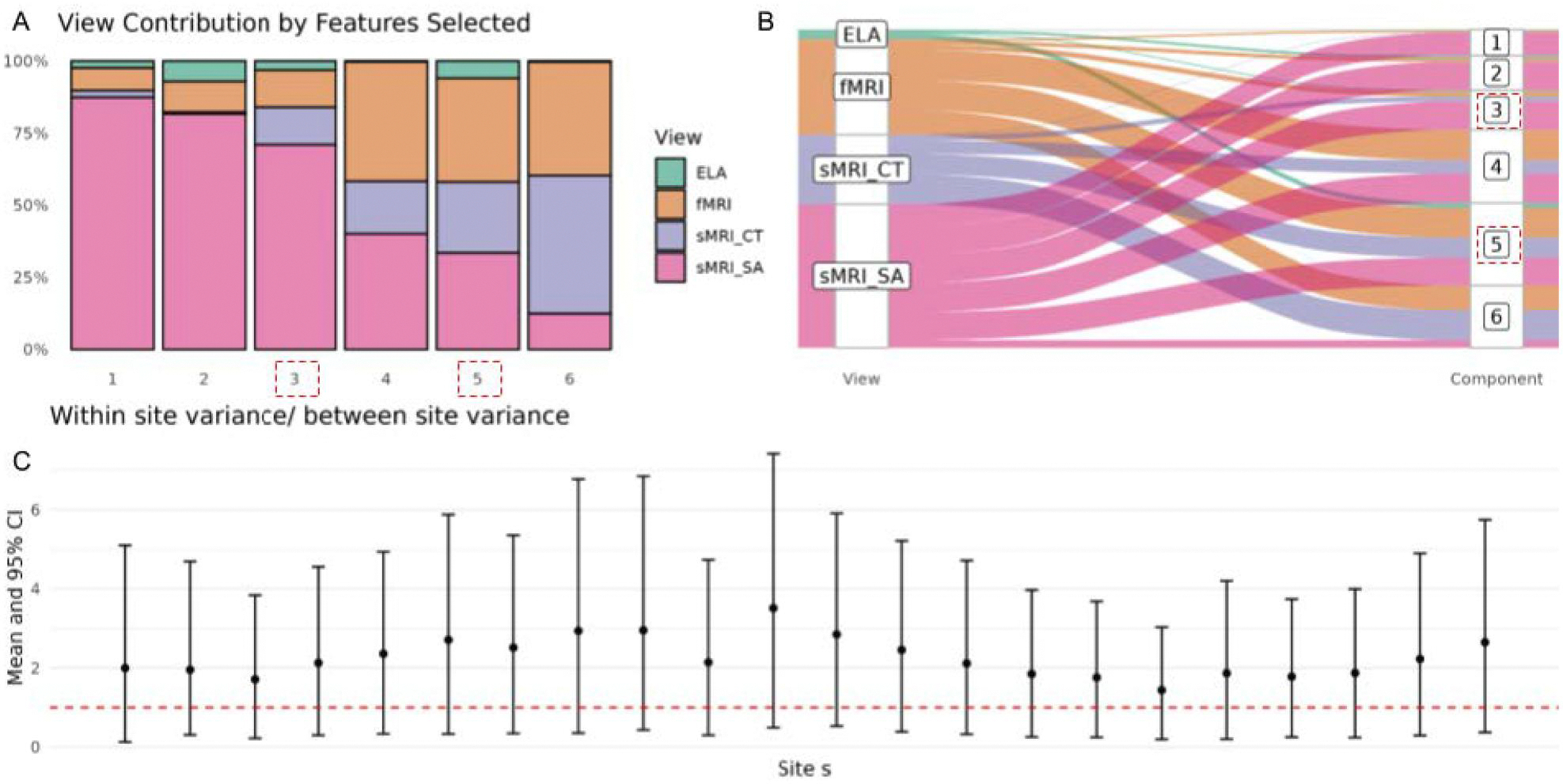
BIPmixed analysis of the ABCD Study dataset with outcome y raw externalizing problems. **Panel A**. View contributions to latent factor components where a contribution is defined as the number of important variables, variables in component I with indicator $\eta_{lj}$ mean marginal posterior probability > 0.5. Views: Early Life Adversity (ELA), functional MRI (fMRI) functional connectivity, and 2 from the structural MRI (sMRI) modality, Cortical Thickness (CT) and Surface Area (SA). Latent factor components 3 and 5, identified as important to the externalizing problems outcome (MPP > 0.5), are highlighted with a dashed box. **Panel B**. Sankey plot important variable mapping from views to latent components with a dashed box around important components. **Panel C**. Within study site variances $\sigma_{\theta_{s}}^{2}$ to between study site variance $\sigma_{\xi }^{2}$ credible intervals, with the dashed line indicating within and between site variance equivalence. Posterior mean (credible interval) for outcome model residual variance $\sigma^{2(0)}$ is 1.255 (1.187, 1.326), and for between study site variance $\sigma_{\xi }^{2}$ is 0.345 (0.118, 1.283).

To identify aspects of early life adversity (ELA) and brain structure and function most strongly associated with externalizing problems, we integrate M=4 views with externalizing problems as the outcome y using BIP and BIPmixed. Figure 3 presents results from applying BIPmixed to the ABCD Study dataset. Each of the four views contributed differently across latent factor components, with contribution defined as the number of variables with marginal posterior probabilities (MPPs) greater than 0.5. Figure 3A shows the proportion of important variables selected across different views. sMRI SA demonstrated notable contributions across all latent components, and ELA represented the least across components. Important components associated with the outcome, as defined by a posterior mean estimate for $y_{l}^{\left(0\right)}$ are emphasized with red dashed boxes, specifically for components l=3,5. Figure 3 B visualizes the mapping of important variables from view to latent factor component using a Sankey plot, again indicating components 3 and 5 as associated with the outcome Externalizing Problems, and showing that while sMRI SA is represented well across all latent factor components, views sMRI CT and fMRI especially co-occur in components 3–6. Figure 3 C depicts the within and between study site variances ($\sigma_{\theta_{s}}^{2}$ and $\sigma_{\xi }^{2}$ respectively) as a forest plot with credible intervals across study sites. The dashed line in Panel C indicates where within-site and between-site variances are equivalent. While credible intervals cover the equivalence point, all posterior mean estimates for $\sigma_{\theta_{s}}^{2}$ are greater than $\sigma_{\xi }^{2}$, suggesting a greater within-site variance than between variance in the externalizing outcome while controlling for covariates and views represented in the latent factor. Posterior estimates for residual variance and between-site variance are 1.255 (1.187, 1.326) and 0.345 (0.118, 1.283), respectively.

Latent factors 3 and 5 are considered important to externalizing problems since ${\overset{ˆ}{\gamma }}_{3}^{\left(0\right)}>0.5$ and ${\overset{ˆ}{\gamma }}_{5}^{\left(0\right)}>0.5$. We describe the top variables, i.e. those with the largest MPP $\overset{ˆ}{\eta }$ in each component. Each component consists of variables across views. For component 3, from the fMRI view, important variables include functional connections from the auditory network to the following: auditory network (self-correlation), cingulo-opercular network, default network, non-Gordon network voxels, and salience network. For the ELA view, top variables were measures of neighborhood safety protocols, parental awareness of the child’s location, avoidant personality problems, and reports of bullying. The sMRI CT view included cortical thickness in the superior precentral sulcus and middle-anterior cingulate gyrus and sulcus. Additionally, from the sMRI SA view, top variables included cortical area in the superior occipital gyrus, and the occipital pole. For component 5, from the fMRI view, important variables included a bidirectional connection between the retrosplenial temporal network and auditory network, retrosplenial temporal network (self-connectivity), salience network (self-connectivity), and a connection from the cingulo-opercular network to the ventral attention network. Top variables from the ELA view were parent-reported measures of neighborhood safety protocols, self-reported neighborhood safety from crime, how often the child and parents eat dinner together, antisocial personality problems, and witnessing violence between adults in the home. The sMRI CT view included structural variables like cortical thickness in the left hemisphere calcarine sulcus, right hemisphere cuneus, and right hemisphere occipital pole. Finally, from the sMRI SA view, top variables were the cortical area in the left hemisphere marginal branch of the cingulate sulcus, the left hemisphere medial occipitotemporal sulcus and lingual sulcus, and the left hemisphere orbital sulci.

In analyzing the impact of covariate inclusion in the Bayesian Integrative Model (BIP) and its mixed variant (BIPmixed) on prediction, we observed a slightly improved performance for BIPmixed when covariates were incorporated directly into the outcome model compared to BIP, where covariates were included as a separate view. Specifically, for the outcome Externalizing Problems R-Score, the Mean (SD) of MSE with covariates was 26.3 (2.66) for BIPmixed compared to 27.4 (3.01) for BIP. Without covariates, MSE was comparable between the methods, with BIPmixed yielding 27.8 (3.36) and BIP yielding 27.8 (2.41). When covariates were modeled as fixed effects, BIPmixed improves accuracy and reduces variability in prediction, as evidenced by an approximate 4% improvement in predictive accuracy (lower test MSE) and a 12% improvement in stability (lower SD of test MSE) for BIPmixed relative to BIP.

The Bayesian Integrative Predictive (BIP) model and its variant, BIPmixed, were implemented in R Version 4.3.0 with Rcpp for efficient computation. Model fitting was conducted on a high-performance computing cluster with 1 CPU core and 32 GB of RAM allocated. For the ABCD Study an average train set size of 5,885.5 observations, 553 variables across four views, across 20 splits, BIP required a mean (sd) 8.69 (0.658) hours and BIPmixed 8.73 (1.97) per MCMC chain, where each chain consisted of 10,000 total iterations, including a burn-in period of 5,000 iterations. BIP and BIPmixed are comparable in average runtime, and BIPmixed is more variable in required runtime.

## 4. Simulation Study

### 4.1. Simulation Scenarios

For each scenario described in the preceding section, we simulate S=20 training and test datasets. We use a method for generating views used in high-dimensional data integration studies (Luo et al. 2016; Chekouo and Safo 2022). We assume 4 views $X=(X^{\left(1\right)},X^{\left(2\right)},X^{\left(3\right)},X^{\left(4\right)})$ where $X^{\left(m\right)}\in R^{n\times p}$. In each $X^{\left(m\right)}$, the first 100 variables form groups where there are 10 main variables, each connected to 9 supporting variables. As a consequence, in each view, there are p-100 singletons. Intraview correlation is $R^{\left(m\right)}=\left(\begin{array}{cc} {\bar{R}}_{100\times 100} & 0\\ 0 & I_{p-100} \end{array}\right)$. ${\bar{R}}_{100\times 100}$ is block diagonal with block size 10, between-block correlation 0, within block 9 × 9 compound symmetry in the supporting variables with elements equal to 0.7^2^, and correlation between a main variable and a supporting variable is 0.7. We assume variable groups contribute to correlation between views by the loadings: $A=(A^{\left(1\right)},A^{\left(2\right)},A^{\left(3\right)},A^{\left(4\right)})$. The first 100 columns $A^{\left(m\right)}\in R^{r=4\times p}$ corresponding to the loading for the first 100 variables aforementioned are sampled from independent and identically distributed (i.i.d.) uniform distribution on [-0.5,-0.3]∪[0.3,0.5], and the main variables’ loadings are multiplied by 2. The remaining p-100 variables are set to 0. Each element of latent factor $U\in R^{n\times r}$ is generated from i.i.d. standard normal. The outcome y in view m=0 is generated with $A^{\left(0\right)}=\alpha =(1,1,1,0)$, i.e. 3 latent components predictive of y. For both the train and test sets, we set p=500 and n= 4, 000 by letting the number of study sites $n_{s}=20$, the number of families per site $n_{f:s}=20$, and the number of individuals per family to $n_{i:f:s}=2$. Study sites are shared across train and test, and families are unique to train and test. Grand μ=1 and random effects ξ and $\theta_{f:s}$ are generated from $\xi_{s}|\mu ,\sigma_{\xi }^{2}\overset{iid}{\sim }𝒩(\mu ,\sigma_{\xi }^{2})$ and $\theta_{f:s}|\xi_{s},\sigma_{\theta_{s}}^{2}\overset{iid}{\sim }𝒩(\xi_{s},\sigma_{\theta_{s}}^{2})$.

For all variables, including the outcome y in view m=0, residual variance $\sigma^{2}=1$. Parameters $\sigma_{\xi }^{2}$ and $\sigma_{\theta_{s}}^{2}$ are different in each simulation scenario. We fix three scenarios describing within-site (i.e. family:site) variance $\sigma_{\theta_{s}}^{2}$ and between-site variance $\sigma_{\xi }^{2}$, where, for a given scenario, $\sigma_{\theta_{s}}^{2}$ is fixed to a specific value across all study sites. In Scenario 1, no random effects are included, with both random effect variances set to 0. Scenario 2 assumes the within-site variance is greater than the between-site variance, with $\sigma_{\theta_{s}}^{2}=1$ and $\sigma_{\xi }^{2}=0.5$. Conversely, Scenario 3 assumes the within-site variance is smaller than the between-site variance, with $\sigma_{\theta_{s}}^{2}=0.5$ and $\sigma_{\xi }^{2}=1$. From $U,\;\alpha ,\;\theta_{f:s},\;$ and $\varepsilon_{i}\sim N\left(0,\sigma^{2}\right),\;$ we generate the outcome y from $y_{i}=U_{i}\alpha +\theta_{f:s}+\varepsilon_{i}$.

### 4.2. Simulation Results

Results include the performance of 4 methods: BIP, BIPmixed, Cooperative Learning, and PCA2Step, a non-integrative comparator, across 3 scenarios that vary in random effects (Table 2). Prediction performance metrics include MSE and variance of predictions $\left(Var(\overset{ˆ}{y})\right)$, and variable selection performance metrics are FPR, FNR, and AUC.

In Scenario 1, BIP achieves the lowest MSE (1.760), closely followed by BIPmixed (1.816) and Cooperative Learning (1.826). PCA2Step (2.054) performs comparably but with a higher variance $\left(Var(\overset{ˆ}{y})=4.091\right)$. Cooperative Learning shows the lowest prediction variance (1.511), suggesting more precise predictions. AUC values for BIP and BIPmixed remain at 1.000, demonstrating perfect variable discriminative ability.

In Scenario 2, where the within-site variance is greater than the between-site variance, BIPmixed delivers the best predictive accuracy with the lowest MSE (2.320) and moderate variance (3.040). PCA2Step (2.822) provides the second-best MSE but exhibits the highest variance (5.265), indicating overfitting to specific views. BIP, while showing a higher MSE (3.242), maintains a low FPR (0.138) and a perfect AUC (1.000). BIP and BIPmixed have 0.000 FNR, meaning that they capture all important variables; however, the non-zero FPR implies that they may falsely flag a variable as important to explaining the outcome of interest. Cooperative Learning continues to excel in variance control (1.338) but suffers from an elevated FNR (0.924). This high FNR implies that Cooperative Learning discards information important to the outcome.

In Scenario 3, where within-site variance is less than between-site, BIPmixed maintains its superiority with the lowest MSE (2.830) and moderate variance (2.596). BIP follows with an MSE of 3.213, outperforming PCA2Step (3.628) and Cooperative Learning (3.363). Variance patterns align with earlier scenarios, where Cooperative Learning retains the lowest variance (1.321). Similar variable selections to Scenario 2 are observed in that BIP and BIPmixed have a FNR of absolute zero and non-zero FPR; whereas, Cooperative Learning has a FPR of zero and FNR of 0.922 (0.024), meaning that BIP and BIPmixed are less conservative than Cooperative Learning in their selection of variables, with the added benefit of retaining all information important to the outcome.

**Figure 4.**
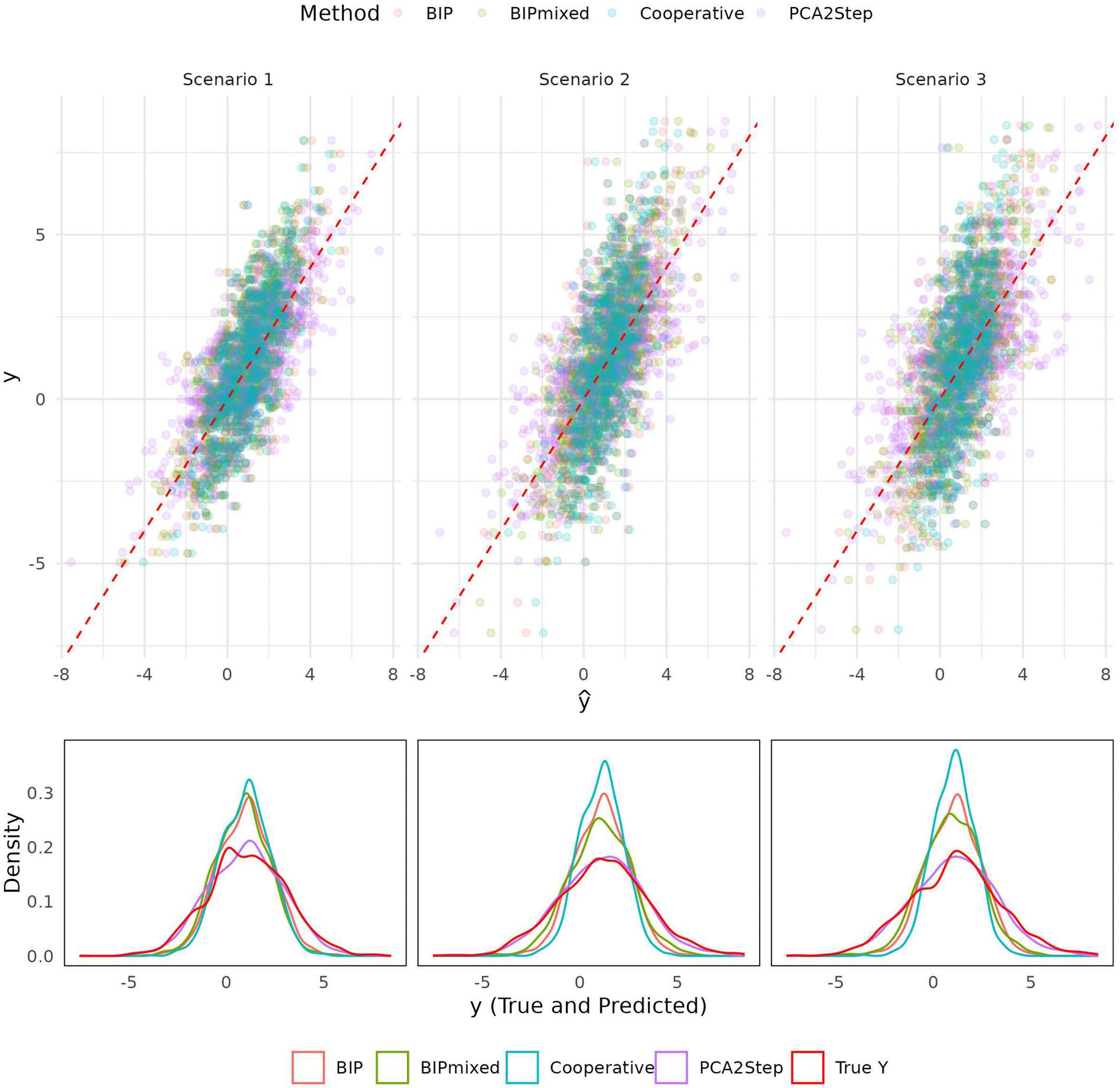
Scatter and density plots of true y vs. predicted $\overset{ˆ}{y}$ for three scenarios by method: BIP, BIPmixed, Cooperative Learning (Cooperative), and PCA 2-step (PCA2Step) from 1 replicate of each scenario. The dashed line represents perfect calibration (slope = 1, intercept = 0) in scatter plots. In Scenarios 2 and 3, BIP and Cooperative depart from the 45-degree line, which is attributable to not accounting for the random effect induced variance. BIPmixed, in contrast, is better calibrated in Scenarios 2 and 3, though PCA2Step reflects y’s variance.

**Figure 5.**
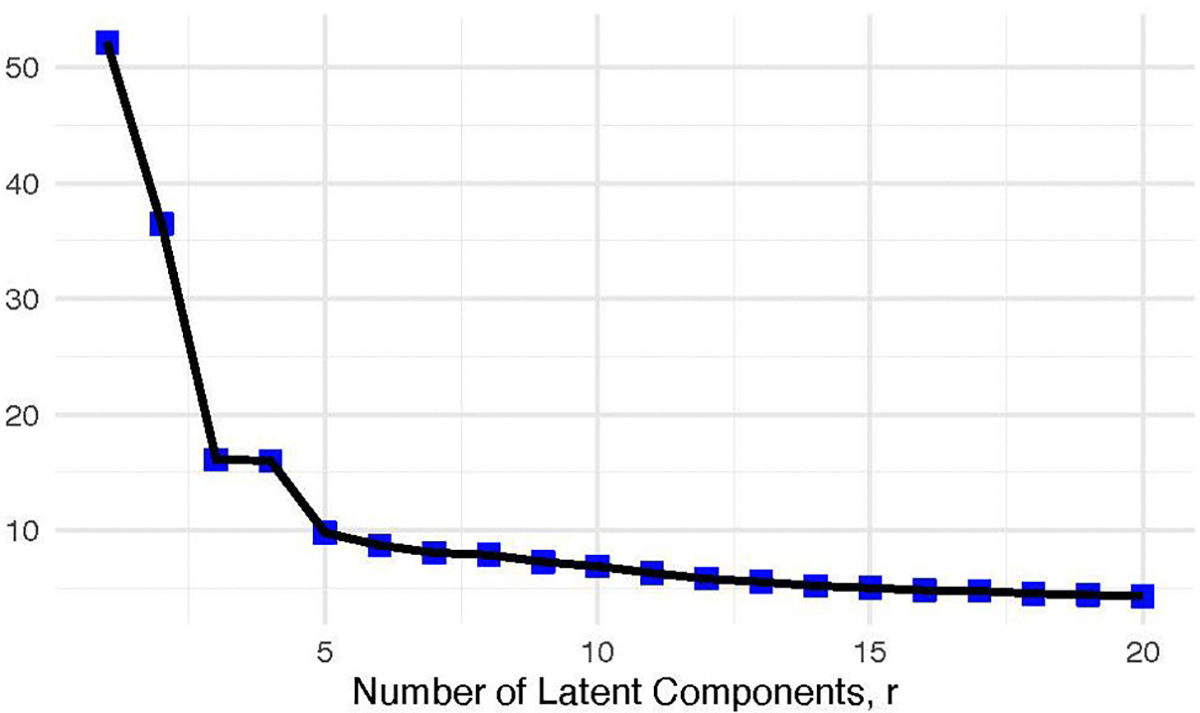
Scree plot of eigenvalues associated with column-wise concatenated ABCD Study train views’ covariance matrix latent components, including Externalizing Behaviors (R-Score) as the outcome. The plots suggest r=6 is a sufficient number of latent components to include in each model, as eigenvalues beyond the 6th component stabilize begin to level off.

Overall, BIPmixed demonstrates superior predictive accuracy in more complex scenarios (2 and 3), while BIP shows competitive performance in a simpler setting (Scenario 1). Figure 4 further demonstrates that BIPmixed exhibits better calibration in Scenarios 2 and 3, as the distribution of predicted outcomes more closely aligns with the true outcome. PCA2Step serves as a non-integrative alternative but suffers from high variance, indicating less stability in its predictions. Cooperative Learning excels in variance control but struggles with variable selection in Scenarios 2 and 3, as reflected by its high FNR, where information important to the outcome is discarded. Meanwhile, BIP and BIPmixed tend to retain variables important to the outcome at the cost of a non-zero FPR. These findings emphasize BIPmixed’s ability to capture hierarchically nested variability, achieving better prediction in such contexts. Consistently high AUC confirms strong variable selection, making it the most reliable method for multiview data similar to that studied.

## 5. Discussion

Simulations reveal performance differences in 4 methods—BIP, BIPmixed, Cooperative Learning, and PCA2Step—across scenarios that vary in random effects. When random effects are negligible (Scenario 1), BIP excels. BIPmixed and Cooperative Learning offer a comparable prediction, demonstrating flexibility even without a strong hierarchical signal. PCA2Step performs well but exhibits high both, indicating potential overfitting when not accounting for multiview structure. In nested scenarios (2 and 3) BIPmixed is the top performer in prediction and variable selection. BIP delivers reliable variable selection, with low FPR and perfect discrimination. Cooperative Learning has low prediction variance but sacrifices variable selection accuracy, as indicated by high FNR. This can be interpreted to mean that Cooperative Learning can discard variables not important to the outcome. BIP and BIPmixed, with the potential of false positives, in these scenarios, include information important to the outcome.

From the ABCD Study data analysis, we found a combination of imaging and ELA variables associated with externalizing problems. The selected ELA factors are consistent with findings from studies on borderline personality disorder and post-traumatic stress disorder, two trauma-based mental health conditions that may exhibit externalization of behaviors (Jowett et al. 2020). Across components identified as predictive of externalizing problems, indicators of neighborhood safety, parental monitoring, and exposure to violence co-occurred with variation in cortical thickness and surface area within cingulate, occipital, and precentral regions, as well as differential connectivity among auditory, salience, retrosplenial, and attention networks. These findings suggest that chronic exposure to environmental stressors and inconsistent caregiving may influence neural circuits involved in sensory integration, salience detection, and emotion regulation. These patterns may represent lower-level neurobiological and environmental processes underlying externalizing behaviors, reinforcing the value of frameworks for understanding the impact of early adversity on development (Kotov et al. 2021; Cuthbert 2022; Conway et al. 2023). Furthermore, the superior prediction performance of BIPmixed when incorporating covariates directly into the outcome model highlights two plausible advantages. First, covariates may carry predictive information that is masked or diluted when treated as a separate view in the latent factor estimation process, aligning with principles seen in wide & deep architectures (Cheng et al. 2016). Analogous to the “wide” portion of such models, fixed and random effects in the outcome model leverage highly predictive covariates to enhance performance, complementing the “deep” component of latent factor estimation. Second, the inclusion of covariates as fixed effects may yield efficiency gains in parameter estimation, reducing prediction variance. This aligns with the concept of efficiency augmentation described in recent literature, which suggests that explicitly modeling covariates improves the precision of latent factor estimates (Huling and Yu 2018). These findings underscore the benefits of integrating covariates into the outcome model to mitigate added variance from parameter estimation, emphasizing their critical role in enhancing prediction accuracy and stability.

These findings suggest that the accommodation of random effects can support prediction performance in large cohort study settings (and others involving hierarchical observations). Regardless, the variables selected by BIPmixed can be interpreted in alignment with a priori known relationships between and within nested observations. Also, the inclusion of covariates in BIPmixed’s outcome model can be helpful in situations where there are strongly predictive covariates available, though this has not been thoroughly investigated in this study and is likely of less utility in behavioral outcome prediction where signal-to-noise tends to be less than, for example, propensity to download mobile apps in the app store (Cheng et al. 2016). A potential benefit of BIPmixed is that including random effects may improve prediction at the tails of the random effect distribution (e.g. families that have an average outcome substantially dissimilar to the overall average) relative to BIP, since BIPmixed predictions are centered at family-specific averages. This may address a general limitation of machine learning methods, which is that they are usually optimized for the central tendency, resulting in predictions that perform less well for certain groups (Malik 2020).

Limitations include that in simpler situations or ones in which the random effects are not explicitly connected to the outcome *y*, BIPmixed has added variance in its predictions, which can reduce prediction performance. Furthermore, a general limitation of the BIP or BIPmixed framework is that the outcome is treated like just another view, which challenges its representation in less signal to noise rich settings (e.g. behavioral outcomes) or in more high-dimensional ones. Moreover, although BIPmixed can incorporate longitudinal data as an additional level in the hierarchical structure (i.e. multiple observations over time nested in an individual), temporal correlation may be distinct from the implied exchangeable correlation. BIPmixed has not been evaluated in the analysis and prediction of simulated or real longitudinal data, which is worth exploring. In addition, exclusion of subjects with missing data may bias results, so a Bayesian or multiple imputation strategy that preserves the multiview structure may enhance this framework’s utility. Moreover, we can implement a strategy that ensures no subjects are excluded from any data view, consistent with the methodology in Chekouo et al. (2017). Given the importance of large-scale longitudinal data like that of the ABCD Study, addressing the aforementioned limitations will improve the benefits of multi-view analyses methods like BIPmixed.

## 6. Conclusion

In conclusion, our study demonstrates the strengths and limitations of 4 multiview learning methods—BIP, BIPmixed, Cooperative Learning, and PCA2Step—across varied scenarios. BIP excels in settings with negligible random effects, while BIPmixed stands out in nested hierarchical contexts for both prediction accuracy and variable selection, leveraging covariates effectively in its outcome model. This highlights the importance of integrating covariates directly into the outcome model to reduce variance and enhance predictive performance, especially for data with hierarchical structures, as seen in the ABCD Study. However, BIPmixed’s benefits diminish in simpler or low signal-to-noise settings, and its reliance on exchangeable correlation structures limits its utility for longitudinal data. Additionally, its treatment of the outcome as another view can challenge its effectiveness in high-dimensional or less informative settings. These findings underscore the need to refine methods like BIPmixed to address limitations, particularly for complex, large-scale cohort data, to further enhance prediction accuracy, variable interpretability, and adaptability across diverse data structures.

## Figures and Tables

**Table 1.** Summary statistics for our sample, reported as mean (SD) or n (%) as appropriate. Race/ethnicity are classified as Native American, Asian, Black, Hispanic/Latinx, Pacific Islander, White, or Other Race Count (%), which can add to more than 100% since endorsement of more than 1 was allowed. Ordinal categorical variables family income in the past 12 months 7.44 (2.31), and parental highest education 17.37 (2.47) are treated as continuous.

Variable	Level	Value

n		7370
Externalizing Problems (Raw)		4.03 (5.44)
Sex (At Birth)	Male	3861 (52.4)
	Female	3509 (47.6)
Age (Months)		119.07 (7.49)
Race/Ethnicity Count (%)	White	5860 (79.5)
	Black	1247 (16.9)
	Native American	225 (3.1)
	Pacific Islander	47 (0.6)
	Asian	475 (6.4)
	Other Race	439 (6.0)
	Hispanic/Latinx	1364 (18.5)
Total Family Income (Past 12 Months)		7.44 (2.31)
Highest Parent Education Completed		17.37 (2.47)
Parent Marital Status	Married	5446 (73.9)
	Widowed	43 (0.6)
	Divorced	576 (7.8)
	Separated	217 (2.9)
	Never married	708 (9.6)
	Living with partner	380 (5.2)

**Table 2.** Performance metrics by Simulation Scenario (S.1, 2, 3) and Method (BIP, BIPmixed, Cooperative): Prediction metrics Mean Square Prediction Error (MSE), variance of predictions $Var(\overset{ˆ}{y})$, and variable selection metrics by Marginal Posterior Probability (MPP) averaged across views False Positive Rate (FPR), False Negative Rate (FNR; threshold = 0.5), and Area Under the Curve (AUC).

Scenario - Method	MSE	Variance	FPR	FNR

1 - PCA2Step	2.054 (0.084)	4.091 (0.281)	NA	NA
1 - BIP	**1.760** (0.075)	2.133 (0.225)	0.125 (0.176)	**0.000** (0.000)
1 - BIPmixed	1.816 (0.087)	2.163 (0.234)	0.131 (0.173)	**0.000** (0.000)
1 - Cooperative	1.826 (0.095)	**1.511** (0.201)	**0.000** (0.000)	0.898 (0.021)
2 - PCA2Step	2.822 (0.196)	5.265 (0.539)	NA	NA
2 - BIP	3.242 (0.236)	2.161 (0.271)	0.138 (0.179)	**0.000** (0.000)
2 - BIPmixed	**2.320** (0.131)	3.040 (0.402)	0.125 (0.157)	**0.000** (0.000)
2 - Cooperative	3.369 (0.289)	**1.338** (0.235)	**0.000** (0.000)	0.924 (0.012)
3 - PCA2Step	3.628 (0.204)	5.210 (0.498)	NA	NA
3 - BIP	3.213 (0.198)	2.202 (0.286)	0.122 (0.165)	**0.000** (0.000)
3 - BIPmixed	**2.830** (0.141)	2.596 (0.319)	0.144 (0.193)	**0.000** (0.000)
3 - Cooperative	3.363 (0.221)	**1.321** (0.193)	**0.000** (0.001)	0.922 (0.024)

MSE indicates prediction accuracy, $Var(\overset{ˆ}{y})$ reflects variability in predictions, and AUC measures discriminative ability by variable selection probability. For BIP and BIPmixed, AUC had a mean (SD) of 1.000 (0.000) across all scenarios. Variable selection metrics FPR, FNR, and AUC do not apply (NA) to PCA2Step, and AUC to Cooperative. BIPmixed shows a lower MSE compared to all other methods in Scenario 2 (2.320) and Scenario 3 (2.830), indicating more accurate predictions in these cases. Second best MSE in Scenario 2: PCA2Step (2.822), and Scenario 3: BIP (3.213). **Bold** text indicates better performance for a given metric.

## Data Availability

Scripts for processing ABCD Study data and performing data analysis and simulation studies in this manuscript are available at github.com/nehera/abcd_multiview. In accordance with the Data Use Agreement, the authors are not able to directly share the Adolescent Brain Cognitive Development (ABCD) Study data used in this study. Eligible researchers who are interested in accessing the ABCD data themselves can request access to the ABCD Study under their own Data Use Agreement/Certification. Further information can be found at the ABCD Wiki (under FAQs: https://wiki.abcdstudy.org/faq/faq.html) and through the NDA (https://nda.nih.gov/user/dashboard/data_permissions.html).

## A. Appendix

### A.1. Posterior Inference

By Markov Chain Monte Carlo (MCMC) sampling, we jointly estimate variable selection indicators $H^{\left(m\right)}={\left(\eta_{lj}^{\left(m\right)}\right)}_{l\in \{1,\ldots ,r\},j\in \left\{1,\ldots ,p_{m}\right\}}$, latent factor indicators $\gamma^{\left(m\right)}={\left(\gamma_{l}^{\left(m\right)}\right)}_{l\in \{1,\ldots ,r\}}$, shared latent factor U, loadings $A^{\left(m\right)}$, fixed and random effects β,ξ and θ. To improve computational efficiency and accelerate MCMC mixing, we employ collapsed Gibbs sampling, where we integrate out the loadings $A^{\left(m\right)}$ before sampling the variable and component indicators, $H^{\left(m\right)}$ and $\gamma^{\left(m\right)}$. Specifically, by marginalizing over $A^{\left(m\right)}$ in the data model, $X^{\left(m\right)}=UA^{\left(m\right)}+E^{\left(m\right)}$, we derive a collapsed conditional distribution for $H^{\left(m\right)}$ and $\gamma^{\left(m\right)}$ that reduces dependency between parameters and improves computational efficiency (Chekouo and Safo 2022). We sample all variable and component indicators $\eta_{lj}$ and $\gamma_{l}$ using Metropolis-Hasting steps. Then, all other parameters are sampled by Gibbs steps. Note, subscript (η) in $A_{\left(\eta \right)}^{\left(m\right)}$ indicates the submatrix of A where $\eta_{lj}=1$.

1. Initialize $\sigma_{j}^{2(m)}=1$ for all m∈{0,1,…,M} and $j\in \left\{1,\ldots ,p_{m}\right\},\;\sigma_{\xi }^{2}=1,\;\sigma_{\theta_{s}}^{2}=0.5\;$ for all $s\in \left\{1,\ldots ,N_{S}\right\},\;\mu =\bar{\tilde{y}},\;\beta ={\left(W^{T}W\right)}^{-1}W\tilde{\tilde{y}}$, and the rest from their priors,
2. After integrating out (i.e., marginalizing over) the loadings $A_{\left(\eta \right)}^{\left(m\right)}$ in the data model $X^{\left(m\right)}=UA_{\left(\eta \right)}^{\left(m\right)}+E^{\left(m\right)}$, a Metropolis-Hastings step is used for each of latent component and variable selection indicators γ,H (Chekouo and Safo 2022),
3. Gibbs steps are used to sample $A_{\left(\eta \right)}^{\left(m\right)},\;\sigma_{j}^{2(m)},\;U$ from full conditionals (Chekouo and Safo 2022),
4. Residualize outcome view on current iteration’s Uα, and Gibbs step outcome model parameters, in order, β (if covariates are available), $\theta ,\;\xi ,\;\sigma_{\theta }^{2},\;\sigma_{\xi }^{2},\;\mu$. Section 7.1.1 below contains details about sampling parameters specific to the outcome view.

#### A.1.1. Outcome Model-Specific Parameter Sampling

Since the site-level random effects follow $\xi |\mu ,\sigma_{\xi }^{2}\sim 𝒩(\mu 1,\sigma_{\xi }^{2}I_{N_{S}})$, then the full conditional distribution of the grand mean μ is as $\mu |\xi ,\sigma_{\xi }^{2}\sim 𝒩\left(m_{\mu },\Sigma_{\mu }\right)$, where $\Sigma_{\mu }={\left(1/\sigma_{\mu }^{2}+N_{S}/\sigma_{\xi }^{2}\right)}^{-1}$ and $m_{\mu }=\Sigma_{\mu }(\sum_{s=1}^{N_{s}}\;\xi_{s}/\sigma_{\xi }^{2})$. The full conditional distribution of the fixed effect vector β is a multivariate normal distribution $\beta |rest\sim 𝒩\left(\mu_{\beta },\Sigma_{\beta }\right)\;$ where $\Sigma_{\beta }={\left(\sigma_{\beta }^{-2}W^{⊤}W+\sigma^{-2}I_{n}\right)}^{-1},\;\mu_{\beta }=\Sigma_{\beta }\left(W^{⊤}(y-Z\theta -U\alpha )/\sigma^{2}\right),\;$ and “rest” indicates conditioning on all other parameters. For site-level random effect vector $\xi ={\left(\xi_{1},\ldots ,\xi_{N_{S}}\right)}^{T}$, for each site s, from families in that site, the full conditional of $\xi_{s}$ is $\xi_{s}|rest\sim 𝒩(\mu_{\xi_{s}},V_{\xi_{s}})$ with $\mu_{\xi_{s}}=V_{\xi_{s}}(\frac{1}{\sigma_{\theta_{s}}^{2}}\sum_{f\in {ℱ}_{s}}\;\theta_{f:s})$ and $V_{\xi_{s}}={\left(\frac{1}{\sigma_{\xi }^{2}}+\frac{n_{s}}{\sigma_{\theta_{s}}^{2}}\right)}^{-1}$ where ${ℱ}_{s}$ is the set of families in site. The full conditional distribution of the family-level random effects θ is also a multivariate normal distribution $\theta_{f:s}|rest\sim 𝒩(\mu_{\theta_{f:s}},\Sigma_{\theta_{f:s}})$ with $\Sigma_{\theta_{f:s}}={\left(\frac{1}{\sigma_{\theta_{s}}^{2}}+\frac{n_{f:s}}{\sigma^{2}}\right)}^{-1}$ and $\mu_{\theta_{f:s}}=\Sigma_{\theta_{f:s}}(\frac{\xi_{s}}{\sigma_{\theta_{s}}^{2}}+\frac{n_{f:s}}{\sigma^{2}}{\bar{\tilde{y}}}_{sf+})$ where $n_{f:s}$ is the number of observations in family f at site s, and ${\bar{\tilde{y}}}_{sf+}$ is the sample mean of the residualized outcome $\tilde{y}=y-W\beta -U\alpha$ over the family f at site s. Full conditionals of site-level variance $\left(\sigma_{\xi }^{2}\right)$ and site-specific family-level variances $\left(\sigma_{\theta_{s}}^{2}\right)$ are defined as: $\sigma_{\xi }^{2}|rest\sim IG(a_{\xi }+\frac{N_{S}}{2},b_{\xi }+\frac{1}{2}\sum_{s=1}^{N_{s}}\;\xi_{s}^{2})\;$ and $\sigma_{\theta_{s}}^{2}|rest\sim IG\left(a_{\theta }+\right.\frac{n_{s}}{2},b_{\theta }+\frac{1}{2}\sum_{f\in {ℱ}_{s}}{\left(\theta_{f:s}-\xi_{s}\right)}^{2}$) with $N_{S}$ number of study sites and $n_{s}$ number of families in site s. The full conditional of the outcome error variance $\sigma^{2}\;$ is $\sigma^{2}|rest\sim IG\left(a_{\sigma }+\frac{N}{2},b_{\sigma }+\frac{1}{2}{\tilde{y}}^{T}\Sigma_{\sigma }^{-1}\tilde{y}\right)\;$ where $\tilde{y}=y-W\beta -Z\theta$ and $\Sigma_{\sigma }^{-1}={\left(U_{\left(\gamma =1\right)}U_{\left(\gamma =1\right)}^{T}+I_{n}\right)}^{-1}$.

### A.2. Hyperparameter Selection: Number of Latent Factor Components

To justify the choice of the number of latent components r used in the BIP and BIPmixed models, we examined the eigenvalues of the concatenated training views’ covariance matrix. The scree plot in Figure 5 indicates that the first six components explain a large proportion of the shared variation, with eigenvalues leveling off thereafter. Accordingly, we set r=6 in the application.
